# Research and application of plasma characteristic models for pulsed laser processing of metal materials

**DOI:** 10.1039/d2ra06709a

**Published:** 2022-12-20

**Authors:** Song Cai, Juan Wang, Qi Li, Wenhao Liu, Guoqi He, Zheying Zhang, Yi Ji

**Affiliations:** School of Intelligent Manufacturing, Wuchang Institute of Technology Wuhan Hubei 430065 China happy9918@sina.com; School of Arts Design, Wuchang University of Technology Wuhan Hubei 430223 China; School of Mechanical Engineering, Hunan University of Technology Zhuzhou Hunan 412007 China; School of Mechanical Science and Engineering, Huazhong University of Science and Technology Wuhan Hubei 430074 China

## Abstract

Plasma characteristic models were established in cylindrical coordinates according to the plasma expansion characteristics of pulsed laser processing of metal materials, mainly including plasma expansion characteristic models and a change rate model for the collisional ionization effect. The plasma characteristics (expansion dimension, expansion velocity, electron density and collision rate) for the pulsed laser machining of a bronze grinding wheel were obtained by using the plasma characteristic models. The results show that the expansion velocity direction can be changed after plasma collision, resulting in particles returning and depositing onto the processed material surface. Plasma spectrum measurements for the pulsed laser machining of a bronze grinding wheel and grinding tests were carried out. Based on the measured spectral data, the plasma electron temperature and plasma electron density were calculated, and the topography of the machined grinding wheel surface was observed, which confirms that black particles can return to cover the grinding wheel surface. Through grinding experiments, it is verified that the returning particles reduce the height of the abrasive protruding binder and block the chip space around the abrasive particles, resulting in reduced grinding performance. The experimental calculation data and numerical simulation results are basically consistent with each other, which not only verifies the correctness and feasibility of the plasma characteristic models but also provides theoretical guidance and process optimization for subsequent research into laser machining of materials.

## Abstract 2

The experimental pulsed fiber laser is manufactured by IPG. The intensity curve for the plasma emission spectra obtained from the material surface is determined.

## Graphical abstract

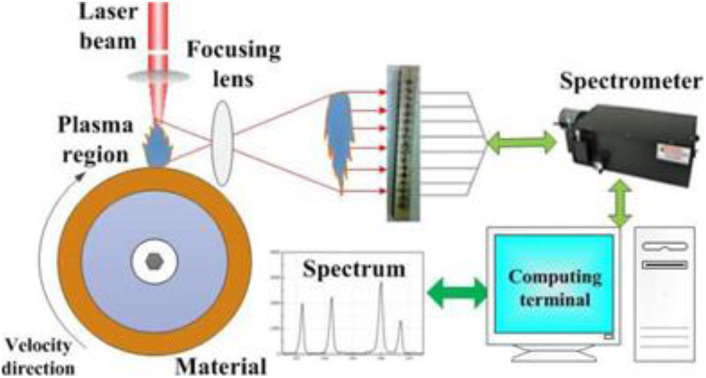

## 1 Introduction

The complex plasma formation mechanism during the interaction of a high-energy pulsed laser with metal materials has been extensively investigated by scholars. Luthra *et al.*^1^ reported various electron impact cross sections, modified the binary encounter Bethe model, and validated the efficacy of the modified model in computing the partial ionization cross sections. Hoffmeister *et al.*^2^ considered the photoionization generated in a system composed of two atoms of different species by a laser field and showed that when the intensity of the laser field is increased, the photoionization process can acquire qualitatively new features in terms of the time evolution and electron emission spectra. Toumi *et al.*^3^ researched the radiative and collisional ionization rate for the emission lines. The above studies show that the plasma formation mechanisms mainly involve photoionization, thermal ionization and collisional ionization; meanwhile, the application of the above plasma models for numerical analysis can be used for theoretical guidance and process optimization. The literature^4^ shows that photoionization and thermal ionization can be studied under the effect of light and heat; however, the essence of the collisional ionization effect is the concentration of the effect of transient changes in the state (momentum, energy, *etc.*) of individual particles with time. The study of individual particle processes is extremely complex, and studies of their change rate models and change rate size have rarely been reported. Related studies^5^ have shown that the plasma collisional ionization effect can affect the expansion characteristics (including dimension and velocity). Therefore, based on the plasma expansion characteristics, the inverse method can be used to study the plasma collisional ionization effect change rate model and change rate size.

Habl L. *et al.*^6^ analysed pulsed neutralization and plasma expansion through two-dimensional particle-in-cell (PIC) simulation and found that the pulse frequency and emission current have a significant effect on the plasma plume potential and the effectiveness of the resulting ion beam neutralization. Mewada *et al.*^7^ reported the chemical doping of graphene (grown on silicon using microwave plasma chemical vapour deposition) with carbon dots and revealed an opportunity for growing graphene directly onto silicon substrates with improved mobility using microwave plasma CVD for various electronic applications. In our research group,^5,8,9^ the plasma expansion equations were established in Cartesian coordinates, and the plasma characteristics were studied, but the above plasma expansion model has many parameters and the solution procedure is extremely complicated. Therefore, based on the accurate description of the plasma expansion characteristics, it is necessary to reduce the parameter variables in the plasma expansion model and establish a new plasma characteristic model with a simpler structure and more convenient solution for studying the collisional ionization effect.

Based on the above research, this paper further analyses the plasma expansion characteristics during pulsed laser processing of materials. The plasma characteristic models in cylindrical coordinates were obtained according to the mathematical and physical distribution laws, which mainly included the plasma expansion models and the change rate model of the collisional ionization effect. The plasma expansion models were used to numerically analyse the plasma expansion characteristics for pulsed laser processing of bronze diamond grinding wheels. The evolution laws for the expansion velocity, expansion dimension and pulse time were obtained. The spatial distribution of the plasma concentration and the evolution of the expansion pressure distribution were plotted. The change rate model for the collisional ionization effect was used to calculate the plasma collisional ionization change rate for pulsed laser processing of a bronze diamond grinding wheel and the mechanism for the surface effect of plasma deposits was described. Relevant experiments were carried out to obtain plasma emission spectral data. Based on the Boltzmann distribution law and the Stark broadening method, the plasma characteristics were obtained and the plasma energy absorption rates were calculated. The topography of the processed bronze diamond grinding wheel surface was observed by a three-dimensional ultradeep field microscope system. Grinding experiments were carried out to observe the surface quality of the grinding wheel after grinding and the effect of plasma deposition on the grinding performance for the laser processing of bronze diamond grinding wheels.

## 2 Plasma characteristic models for nanosecond laser processing of materials

Plasma characteristic models consist mainly of the plasma expansion models and the change rate model of the collisional ionization effect. The following assumptions were made before the model was established:^9–11^ because of the complexity of researching the laws of motion of individual particles during nanosecond pulsed laser processing of materials, the plasma can be considered an ideal spherical gas in practical studies, satisfying local thermal equilibrium conditions. In the expansion of plasma spherical gases, the pressure gradient is large and the expansion velocity is fast. The plasma expansion involves a continuous process during the pulsed irradiation time, which is approximately consistent with the motion characteristics of the continuous fluid, with an exponential decrease in concentration and expansion pressure with increasing diffusion distance.

### 2.1 Plasma characteristic models

**Fig. 1:**
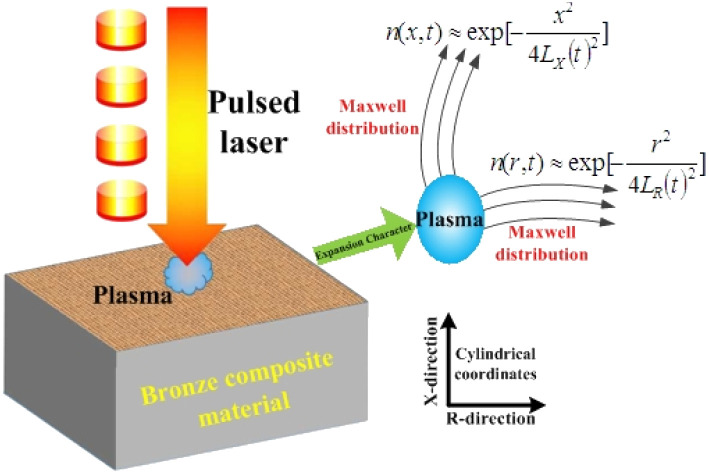
The diagram of the plasma concentration spatial distribution.

The centre coordinates were set as the position of the laser spot. A schematic diagram of the plasma concentration spatial distribution is shown in Fig. 1. A plasma is formed during the laser processing of materials. In the *X*-direction, plasma expansion is prevented by the material, and the particles expand in the direction opposite to the laser beam. In the *R*-direction, the splashed particles have an initial velocity consistent with the rotation direction of the machining platform. In the *X*- and *R*-directions, the particles collide violently, producing a constant velocity over a small area.

Based on the above assumptions and analysis, research on the velocity laws for individual particles is complicated. However, within a certain spatial range, all particles can be considered ideal spherical gases, with particles entering and leaving this range with approximately the same velocity, close to the dynamic equilibrium state, in accordance with the Maxwell velocity distribution characteristics.^12,13^ The relationship is set as follows:1
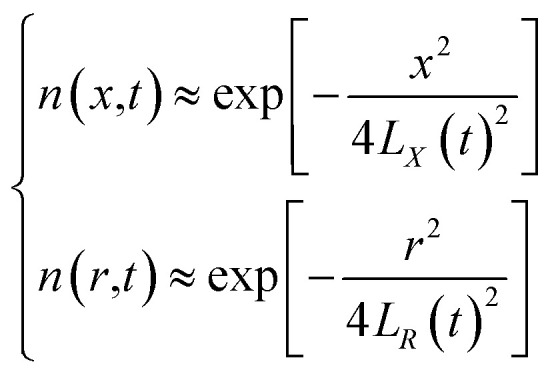


From the above characteristics for the plasma distribution in the *X*- and *R*-directions, it is known that the plasma concentration spatial distribution equations should contain the characteristic term describing the Maxwell velocity distribution. Therefore, the plasma distribution equation in the column coordinates is established as follows:2
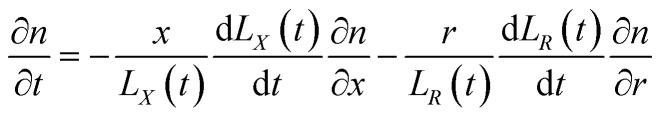


The left side of the equation is the change rate in the number of particles, and the right side of the equation is the change rate due to the change in the spatial position of the particles in the *X*-direction and *R*-direction satisfying the Maxwell velocity distribution factor term. Therefore, this equation can be solved to obtain (see Appendix):3
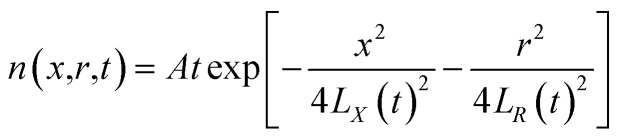


The integration in cylindrical coordinates can be obtained as follows:4
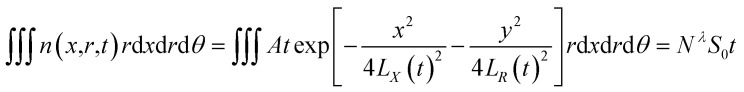
where, 
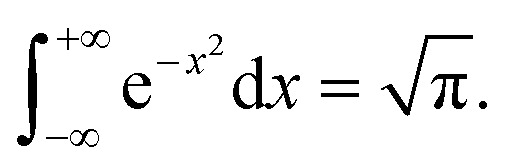
 The expressions for *A* and *N*^*λ*^ can be found from the literature^14^ (see Appendix):5
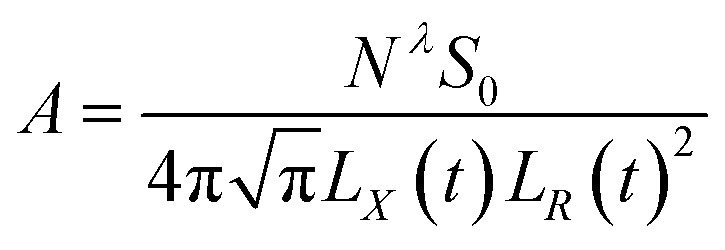
6
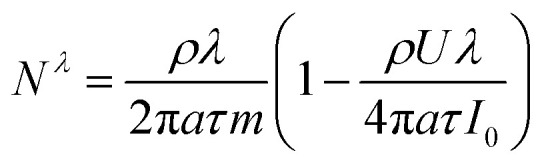


By substituting eqn (5) and (6) into eqn (3), one can obtain the plasma concentration spatial distribution equation:7
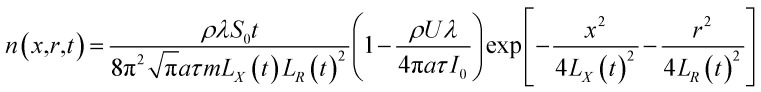


Considering the equation *P* = *nk*_b_*T* under ideal gas conditions, the plasma expansion-induced external pressure equation is obtained as follows:8
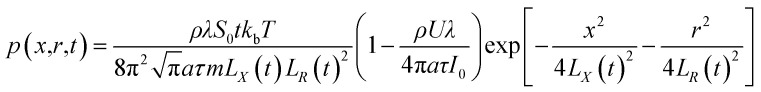


The above equations are based on satisfying the conditions of local conservation of mass and momentum, and the local conservation law is much stricter than the overall mass conservation law and momentum conservation law, which can better reflect the actual processing situation. In cylindrical coordinates, the local mass conservation law can be expressed as follows:^15^9
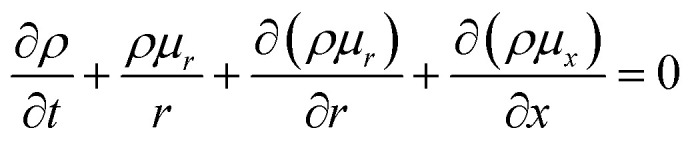
where *ρ* = *nm*; *μ*_*r*_ is the velocity parallel to the material surface, which is a function of *r* and *t*; *μ*_*x*_ is the velocity perpendicular to the material surface, which is a function of *r* and *t*. By substituting the spatial distribution of the plasma concentration into eqn (9), we can obtain10
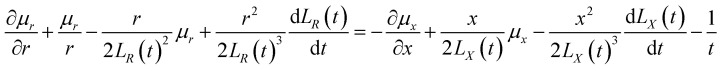


Eqn (10) requires both expressions on either side of the equal sign to be constant; so, both sides of the equation must be a function of time *t*(*q*(*t*)), and we can obtain11
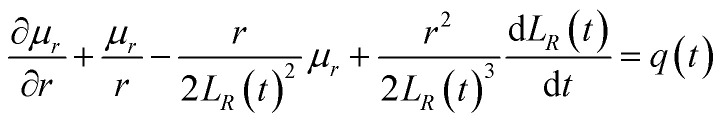
12
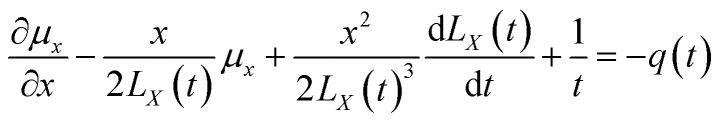


Eqn (11) and (12) can be solved to obtain (see Appendix)13
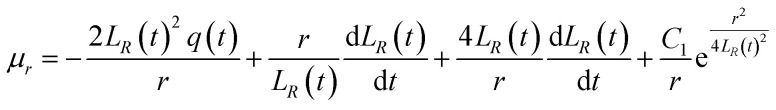
14
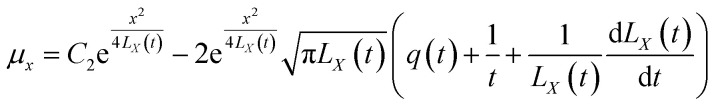


Substituting the boundary conditions gives15
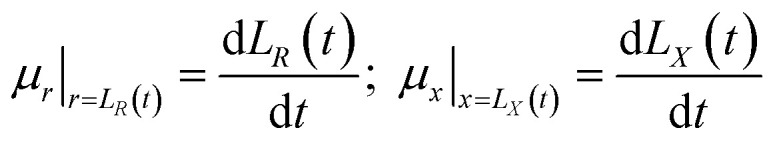


We can obtain16
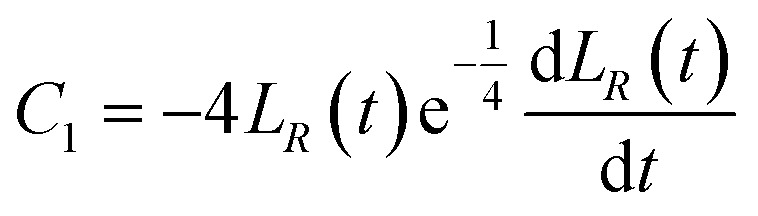
17
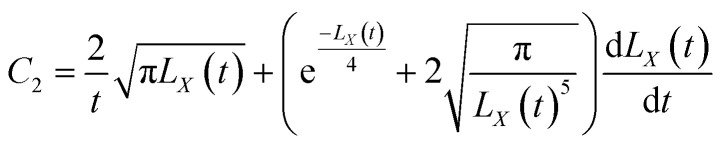


By substituting *C*_1_ and *C*_2_ into the expressions for *μ*_*r*_ and *μ*_*x*_, we can obtain18
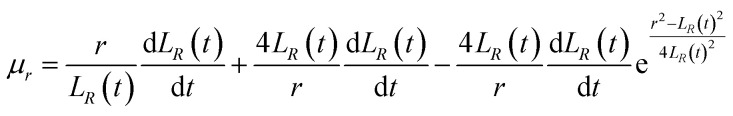
19
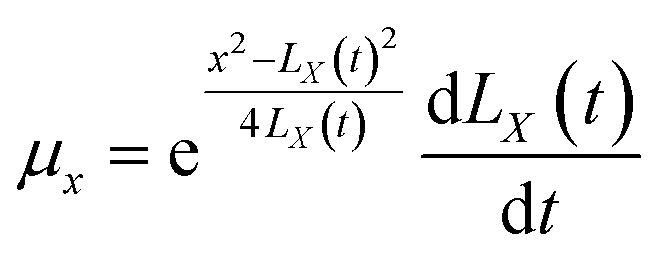


In cylindrical coordinates, the conservation of momentum equations are as follows:^16–18^20
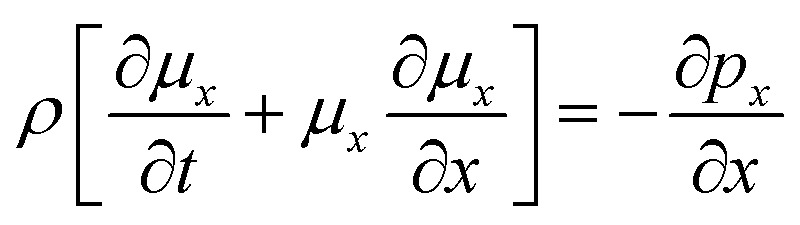
21
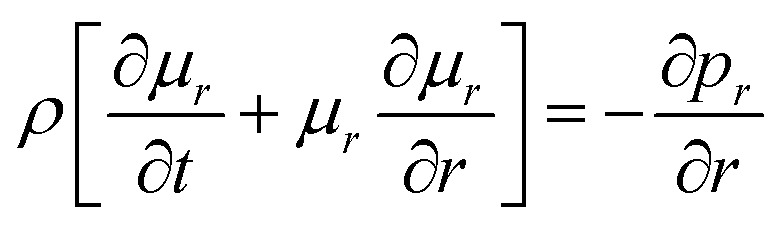


By substituting *x* = *L*_*X*_(*t*) and *r* = *L*_*R*_(*t*) into eqn (20) and (21), the plasma expansion kinetic equations are obtained as follows (see Appendix):22
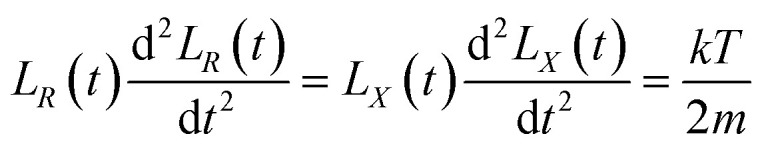
where the initial velocity is given by:^19^23
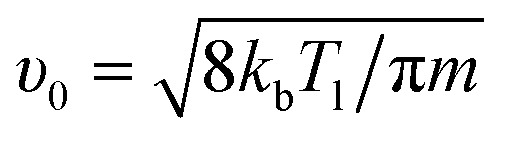


### 2.2 Change rate model of the plasma collisional ionization effect

Under the condition of a small optical thickness, plasma collisional ionization is an important process. At low temperature and low density, the collisional ionization in a plasma mainly involves two-body collisional ionization, where the electron radius is negligible with respect to the neutral particle, and the collision cross section between the electron and neutral particle is given by *σ* = π*r*^2^. The average molecular free path is calculated to be *λ* = 1/*nσ*, where *n* is the plasma electron density, *σ* is the particle collision cross section and 
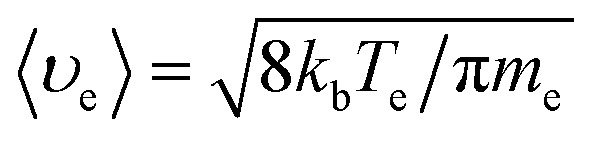
 is the average plasma electron velocity. According to the two-body collision frequency equation, after substituting the equation for the average free path into the plasma concentration spatial distribution equation, the change rate equation for the plasma collisional ionization is obtained as follows (see Appendix)24
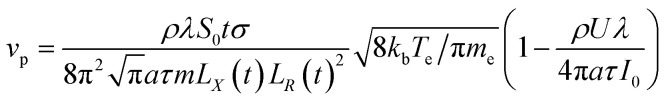


In summary, the plasma expansion characteristic models (mainly including the plasma concentration spatial distribution eqn (7) and the plasma expansion-induced external pressure eqn (8)) and the plasma expansion kinetic eqn (22) are established in cylindrical coordinates. Coupled with the two-body collision frequency equation, the change rate equation for the plasma collisional ionization (24) is finally obtained. Compared with the model reported in the literature,^8^ the above models have a simpler structure, fewer computational parameters, and a more convenient procedure for obtaining the solution.

## 3 Research on plasma expansion characteristics and deposition effect of pulsed laser processing of bronze diamond grinding wheel

High-power lasers are widely used in dressing bronze diamond grinding wheels. The pulsed laser dressing of CBN grinding wheels and bronze diamond grinding wheels with good dressing quality was studied by related researchers.^20–22^ However, during the laser dressing process, the study of the plasma expansion characteristics is complicated, and research into its deposition effects is even less reported. In this section, the plasma expansion characteristics model in cylindrical coordinates is used to numerically analyse the plasma expansion characteristics for pulsed laser processing bronze diamond grinding wheels, calculate the change rate for the plasma collisional ionization, and elaborate the mechanism for the plasma deposition effect.

### 3.1 Plasma expansion characteristics

According to the plasma expansion kinetic eqn (22), the plasma expansion kinetic difference equation is established by the finite difference method as follows:25
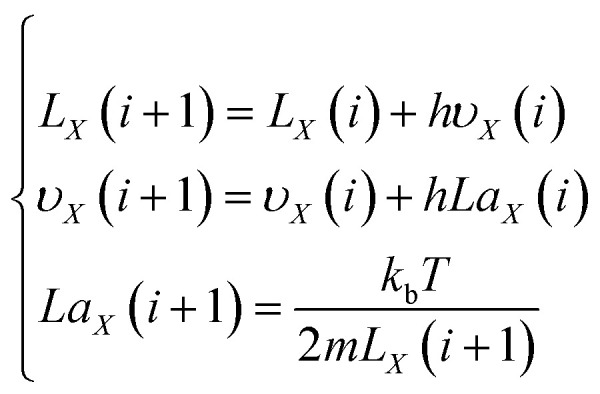
26
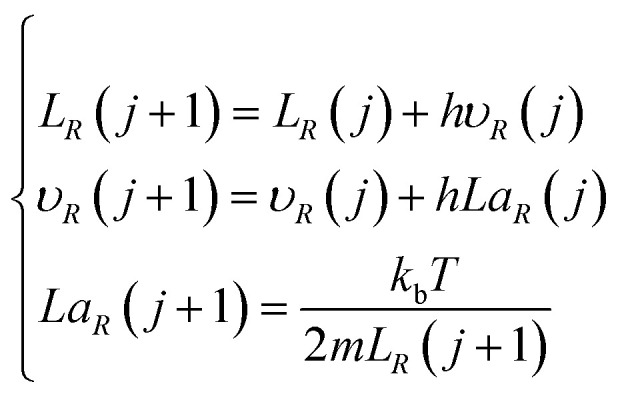


To verify the correctness of the model, the plasma electron temperature is set in the numerical simulation to be approximately the same as the experimentally obtained electron temperature of approximately 8500 K.

#### 3.1.1 Plasma expansion dimension and expansion velocity

The plasma expansion process can be approximated as an ideal gas. The initial plasma expansion dimension is approximately equal to the molecular average free path range in the *X*- and *R*-directions. Based on the above analysis and the reported literature,^14^ it is shown that the initial expansion dimension in the *X*- and *R*-directions can be approximated as 10^−6^ m, and the initial velocity in the *X*-direction can be calculated from eqn (23) to be approximately 968 m s^−1^. The initial velocity in the *R*-direction is approximately 1.530 m s^−1^, which is consistent with the rotational velocity of the working platform in the experiment, and the initial acceleration is approximately 645 × 10^9^ m s^−2^.

**Fig. 2:**
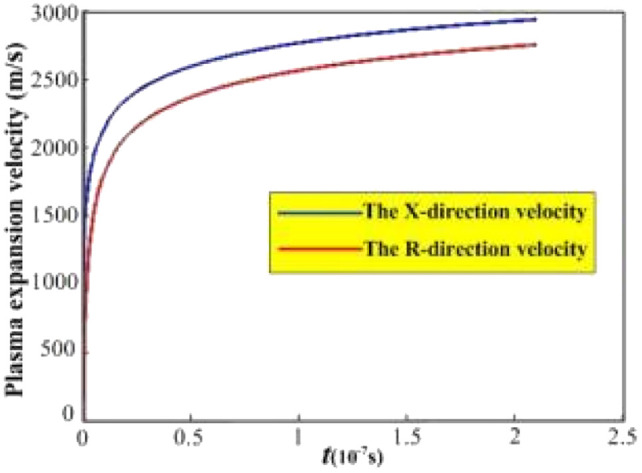
Evolution curve of plasma expansion velocity.

Fig. 2 shows the evolution curve for the plasma expansion velocity, with the *X*- and *R*-directions indicated in blue and red, respectively. Both curves shown in Fig. 2 show a nonlinear increase. In the range of 0–25 ns for the fast-rising section of the curve, the maximum velocity reaches 2900 m s^−1^ and 2600 m s^−1^ in the *X*- and *R*-directions, respectively. During the expansion time of 25 ns–210 ns, the expansion rate tends to be flat and increases less. From the numerical simulation, it can be deduced that for a laser irradiation time of approximately 25 ns, the particle expansion velocity reaches a maximum value, and the particles collide violently with each other, leading to an increase in the particle collisional ionization effect with the plasma concentration reaches its maximum value. Subsequently, the expanding volume of the plasma leads to energy consumption, resulting in a slow increase in velocity. Eventually, the plasma concentration will decrease with the recombination of electrons and ions.

**Fig. 3:**
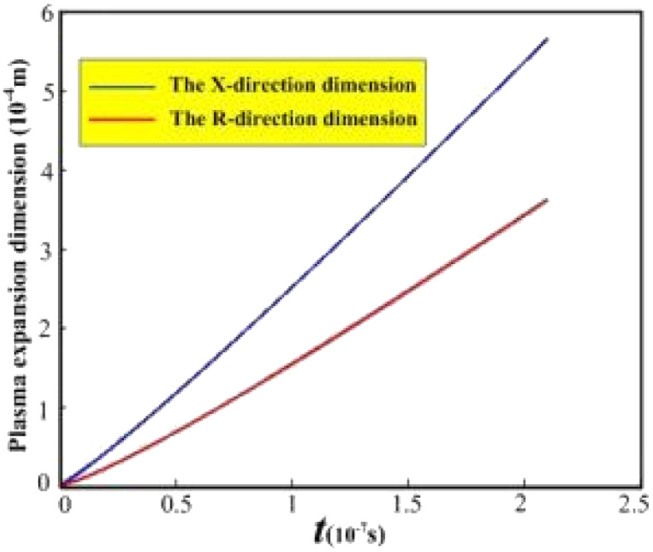
Evolution curve of plasma expansion dimension.

The evolution curve for the plasma expansion dimension is shown in Fig. 3. In the *X*- and *R*-directions, the plasma expansion dimension gradually increases with increasing pulsed laser irradiation time, showing a linear evolution law. Fig. 3 shows after the pulsed laser is stopped, the plasma expansion dimensions are approximately 5.5 × 10^−4^ m (*X*-direction) and 3.3 × 10^−4^ m (*R*-direction), respectively. This is mainly due to the continuous expansion of the plasma formed by the material absorbing the laser energy during the irradiation time of the pulsed laser, which leads to a gradual increase in the expansion dimension.

#### 3.1.2 Plasma concentration spatial distribution and expansion-induced external pressure

By substituting the numerical simulation results and the calculated parameters given in Table 1 into the plasma concentration spatial distribution eqn (7) and the plasma expansion-induced external pressure eqn (8), the following equations can be obtained:27
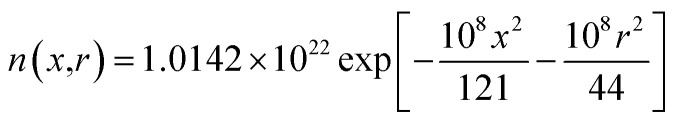
28
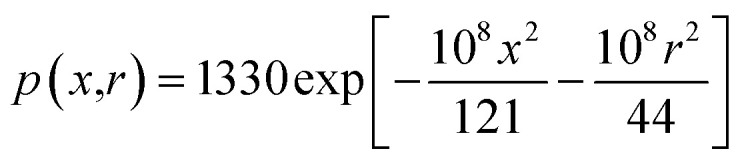


**Table 1:** Plasma calculation parameters^9^

Name	Symbol	Numerical value and units	Name	Symbol	Numerical value and units
Electron temperature	*T*	8500 K (0.73 eV)	Laser power density	*I*_0_	3.359 × 10^8^ W cm^−2^
Gasification temperature	*T*_l_	2770 K	Spot size	*S*_0_	1.134 × 10^−9^ m^2^
Ionization energy	*U*	7.631 eV	Pulse time	*τ*	2.1 × 10^−7^ s
Atomic mass	*m*	1.038 × 10^−25^ kg	Refractive index	*a*	1.18
Laser wavelength	*λ*	1064 nm	Solid phase density	*ρ*_s_	8620 kg m^−3^
Photon energy	*hν*	1.160 eV	Average charge	*Z*	2
Boltzmann constant	*k*_b_	1.381 × 10^−23^ J K^−1^	Planck constant	*H*	6.626 × 10^−34^ J S

**Fig. 4:**
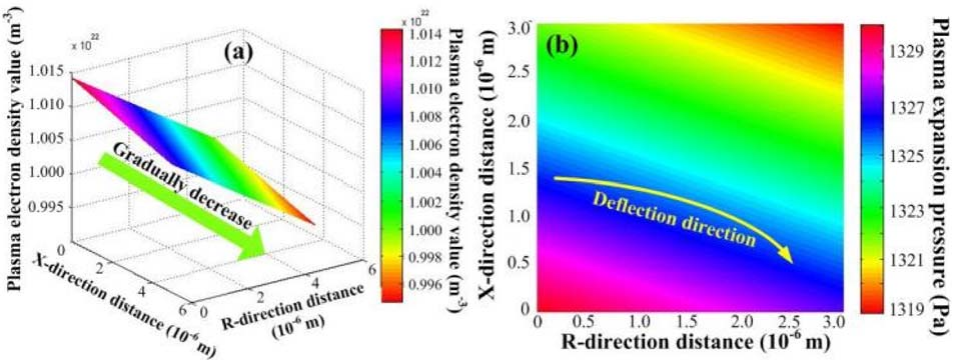
Plasma electron concentration and expansion external pressure distribution. (a) Plasma electron concentration; (b) pressure distribution.

According to eqn (27) and (28), the plasma concentration spatial distribution and expansion-induced external pressure are found to decrease gradually with increasing distance, showing a high inside and low outside distribution law. As shown in Fig. 4, the maximum plasma concentration and maximum expansion-induced pressure is approximately 1.014 × 10^16^ cm^−3^ and 1330 Pa, respectively, at the material surface. At the same time, the initial expansion velocity of the splashing particles in the *R*-direction leads to a deflection of the plasma expansion direction.

After the plasma was formed by pulsed laser processing of bronze material, the electrons and neutral particles in the plasma collided with each other in a high-temperature and high-pressure environment, causing the neutral particles to ionize and resulting in an increase in the plasma electron density. According to the above analysis and the Saha equation,^23,24^ the ionization degree is approximately 0.7 and the maximum plasma electron density is approximately 1.724 × 10^16^ cm^−3^ when the plasma electron temperature of approximately 8500 K under the condition of considering only the first-order ionization degree. At the same time, the plasma expansion-induced external pressure is low, and the plasma expansion dimension is small and the plasma eventually disappears into the atmosphere after expanding for some distance.

### 3.2 Plasma collisional ionization change rate

For monatomic gases, at a higher the gasification temperature, the corresponding ionization potential is usually lower. In this case, even if the concentration of particles is not high after bronze vapourization after laser processing, there are still many free electrons (approximately 1.014 × 10^16^ cm^−3^) in the ionized bronze vapour when the vapour pressure is higher than the ambient pressure (approximately 1330 Pa). Collisional excitation occurs when free electrons collide with copper atoms and release bound electrons. During the laser dressing process, plasma is considered an ideal gas, and its collisional characteristics are dominated by two-body collisions at low temperatures and low densities.

**Fig. 5:**
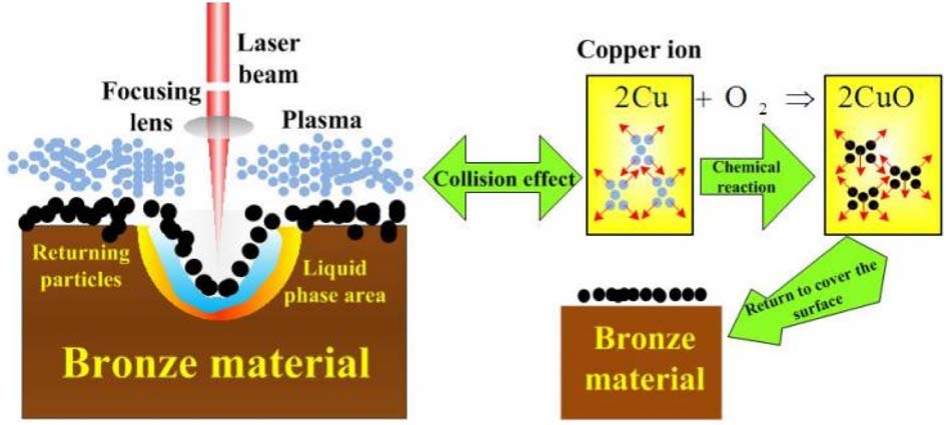
Research on the mechanism of plasma deposition effect.

The plasma is dominated by copper atoms with an atomic radius of 0.13 nm. The collision cross section of electrons with neutral particles is calculated to be *λ*_en_ = 0.002. The average velocity of electrons 〈*υ*_e_〉 is 6.055 × 10^5^ m s^−1^. Ultimately, *v*_p_ = 3.187 × 10^8^ s^−1^ is obtained from the change rate equation for the plasma collisional ionization (24). The above numerical analysis shows that the plasma collides with each other at a high frequency during the expansion process, and the expansion velocity direction will change after the collision. In the high-temperature environment, the combination of copper ions and oxygen will lead to the generation of black particles of copper oxide that return to the material surface. The mechanism for the whole process is shown in Fig. 5.

## 4 Experiments for the nanosecond laser processing of materials

The threshold of laser processing materials is mainly determined by the laser wavelength and pulse width. The power density threshold of the laser processing of materials can be determined by eqn (29).^25^29
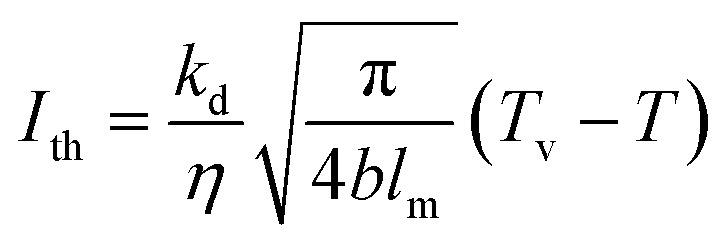


To verify the correctness of the plasma expansion characteristic model, experimental measurements for plasma emission spectra of pulsed laser processing of bronze diamond grinding wheels were carried out.

### 4.1 Experimental equipment and materials

**Fig. 6:**
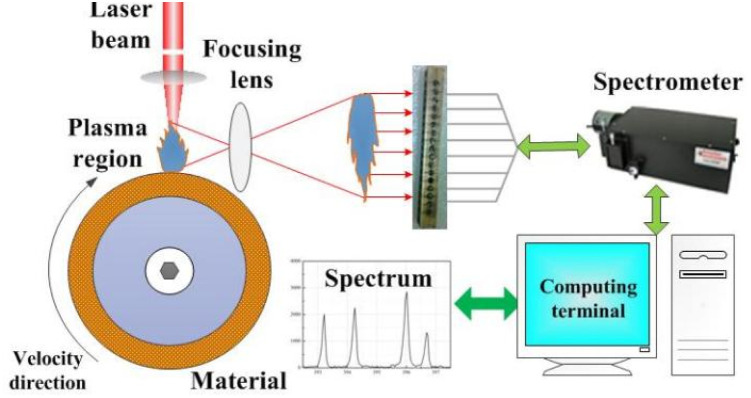
Schematic diagram of pulsed laser processing materials.

As shown in Fig. 6, the experimental pulsed fiber laser (model: YLP-1/120/50/50-HC) is manufactured by IPG. The laser pulse energy can reach 1 MJ, beam quality *M*^2^ is 1.5, energy stability is about 2%∼5% (1064 nm), the laser wavelength is 1064 nm, the average output power is 0–50 W, the pulse repetition rate is 50–150 kHz, the output pulse shape is Gaussian distribution, the laser pulse width is 210 ns, and the diameter of the focused spot is 38 μm.

A SpectraPro-2300i spectrometer equipped with charge-coupled devices with parameters of 1340 × 400 pixels 400F was used to acquire the spectral signals. The central wavelengths were measured to be 520 nm and 580 nm, respectively, and the widths were measured to be approximately 130 nm. The data were stored in a computer, and the obtained plasma emission spectra were displayed on a computer screen after processing. Bronze material with a thermal conductivity of 41.9 W m^−1^ K^−1^ was used as the experimental material, the laser absorption was 0.38, the thermal diffusivity was 0.14 cm^2^ s^−1^, the vapourization temperature was 2770 K and the initial temperature was 300 K. The laser power density threshold was determined to be approximately *I*_th_ = 1.411 × 10^7^ W cm^−2^ by using eqn (29). Therefore, the laser power density threshold in the experiment needs to exceed the processing threshold for the bronze material before subsequent experiments can be conducted.

### 4.2 Research on plasma characteristics of laser processing bronze diamond grinding wheel

In the experiment, the laser power was 40 W, the laser pulse frequency was 20 kHz, the rotation speed was 300 rpm, and the laser power density was 3.359 × 10^8^ W cm^−2^. The delay time for the spectrometer was approximately 2 μs, and the pulse integration time was approximately 25 μs. The above parameters indicate that the laser outputs 2 × 10^4^ laser pulses per second, that is, the duration of each pulse is approximately 5 × 10^−5^ s (50 μs). It has been reported in the literature^7^ that the duration of the plasma cycle is usually 10 μs. Therefore, the plasma generated by a laser pulse will not affect the plasma generated by a subsequent pulse in this experiment.

In the experiment, twenty pulses were selected continuously. That is, after approximately 1 × 10^−3^ s, the data acquisition was stopped, and then the acquired data were stored in a computer and integrated to generate the spectra. Based on the spectral data, the Boltzmann plot method and the Stark broadening method were used to calculate the plasma electron temperature and plasma electron density, respectively,^26–28^ to finally verify the correctness of the plasma expansion model.

#### 4.2.1 Plasma electron temperature

**Fig. 7:**
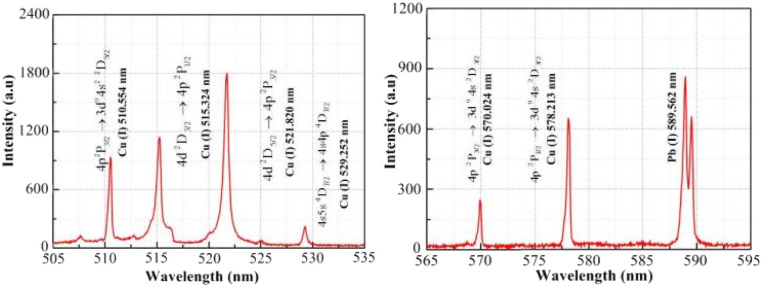
Intensity curve of the plasma emission spectrum.

The intensity curve for the plasma emission spectra obtained at a distance of approximately 0.12 mm from the material surface is shown in Fig. 7. The intensity values for the six atomic spectral lines of copper (Cu(i) 510.551, 515.324, 521.820, 529.252, 570.024 and 578.213 nm) are marked in Fig. 7. The Boltzmann plot method was used to calculate the plasma electron temperature, which was fitted to obtain the straight line slope. Table 2 shows the copper atomic spectral parameters required for the calculations.^8^

**Table 2:** Spectroscopic parameters of Cu atoms

Line (nm)	Statistical weight		Transition probability *A*_*pq*_ (s^−1^)	Excitation energy *E*_*p*_
	*g*_*p*_	*g*_*q*_		
510.554	4	6	2.0 × 10^6^	3.830
515.324	4	2	6.0 × 10^7^	6.191
521.820	6	4	7.5 × 10^7^	6.192
529.252	8	8	1.09 × 10^7^	7.750
570.024	4	4	2.4 × 10^5^	3.820
578.213	2	4	1.65 × 10^6^	3.790

**Fig. 8:**
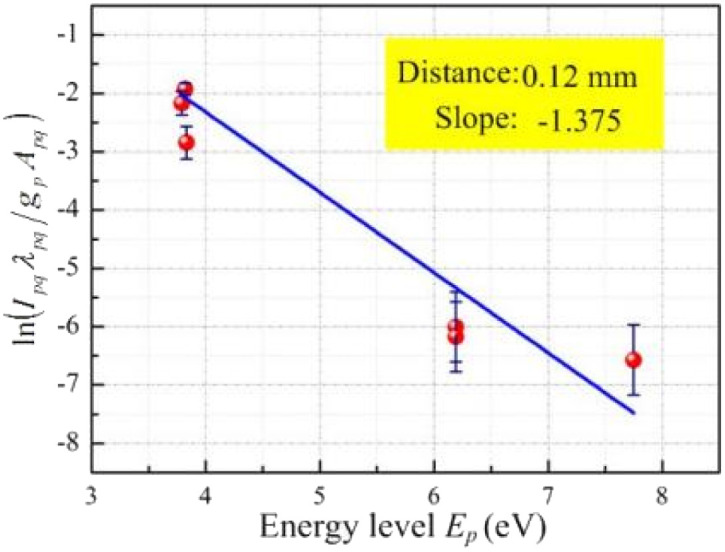
The Boltzmann plot of laser processed copper.

The Boltzmann plot was determined by linear fitting as shown in Fig. 8, and the slope value for the line was determined to be approximately −1.375. The Boltzmann plot method^28^ shows that *T*_e_ = −1/(*k*_b_*X*_S_), where *X*_S_ is the value of the slope of the straight line. By substituting the slope value (*X*_S_) into the temperature equation, the plasma electron temperature was calculated to be approximately 8438 ± 840 K. The calculation error is approximately 10%, which is mainly attributed to the measurement error, parameter error, and data fitting error. The experimentally obtained plasma electron temperature was determined to be in general agreement with the numerical calculations, providing a basis for subsequent validation of the plasma expansion model.

#### 4.2.2 Plasma electron density

**Fig. 9:**
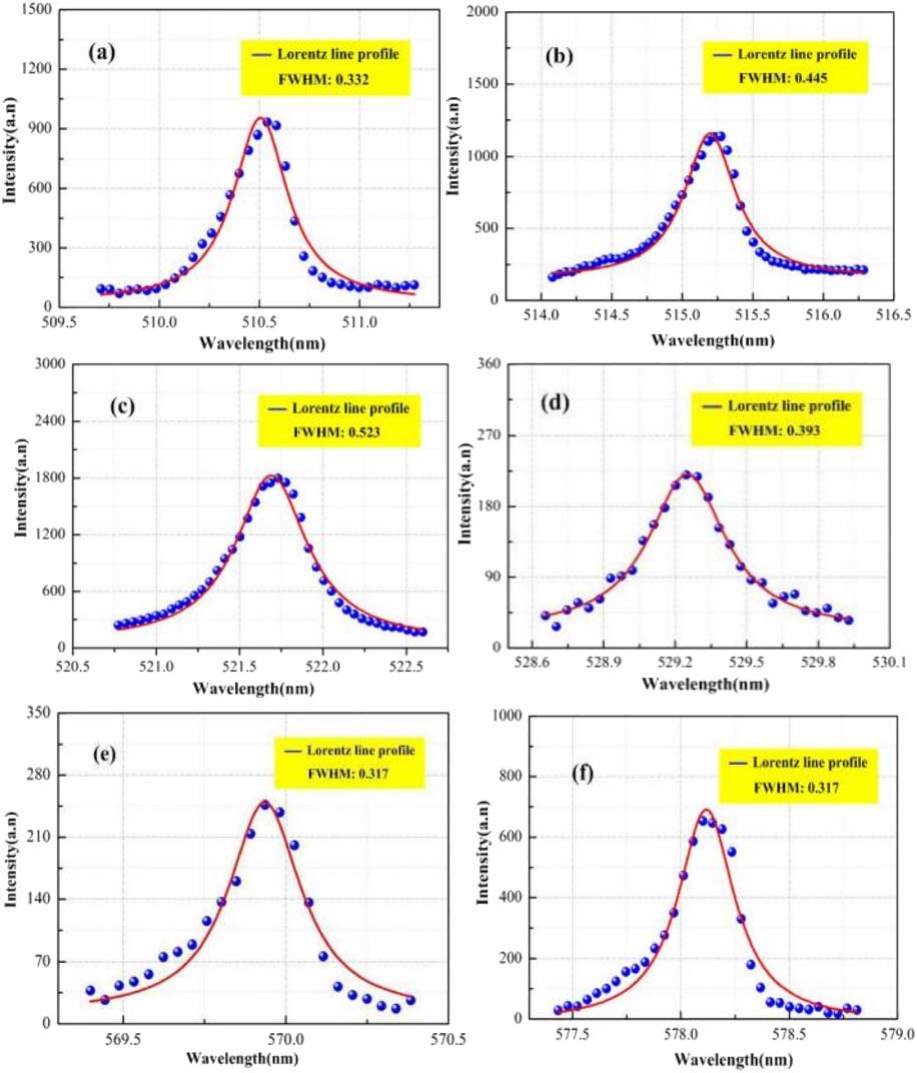
Peak intensity of copper atom spectral line. (a) 510.554 nm; (b) 515.324 nm; (c) 521.820 nm; (d) 529.252 nm; (e) 570.024 nm; (f) 578.213 nm.

The plasma spectral data were obtained based on experimental measurements. Fig. 9 shows that the plasma spectrum mainly consists of continuous background spectra and copper atomic emission spectra. At present, the plasma electron density is mainly calculated by means of the spectral line broadening parameter. In practice, it has been found that spectral line broadening is affected by many factors, including Stark broadening, Doppler broadening, resonance broadening, self-absorption broadening, and the effect of the measuring instrument on spectral line broadening. It has been reported in the literature^29,30^ that Doppler broadening, resonance broadening and self-absorption broadening have a small effect on spectral line broadening and can generally be neglected. Therefore, spectral line broadening for calculating the plasma electron density is mainly performed using Stark broadening and instrumental broadening. Six atomic spectral lines (Cu(i) 510.551, 515.324, 521.820, 529.252, 570.024 and 578.213 nm) were selected to calculate the plasma electron density in the experiment. The full width at half maximum (FWHM) for the peak intensity of the spectral lines can be obtained by fitting the spectral line data with a Lorentz function, as shown in Fig. 9(a)–(f), with the above six atomic lines having a FWHM of being 0.332, 0.445, 0.523, 0.393 0.271 and 0.317, respectively, where, the final FWHM of the spectral line (Δ*λ*_1/2_) is the FWHM of the peak intensity of the spectral line minus the effect of the measuring instrument on the spectral line broadening (approximately 0.15 nm), which is substituted into the Stark broadening formula to obtain the plasma electron density.

**Fig. 10:**
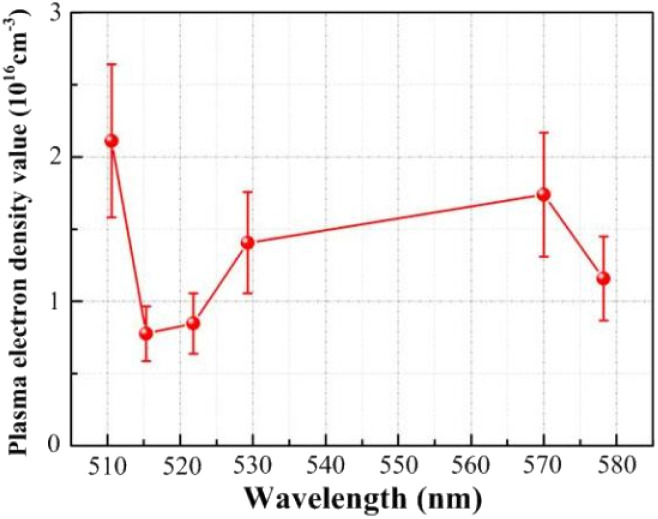
Distribution curve of plasma electron density.

Based on the FWHM (Δ*λ*_1/2_) and the Stark broadening method measured by the six spectral lines, the distribution of the plasma electron density is shown in Fig. 10. Based on the spectral data obtained for Cu(i) 510.551 nm, Cu(i) 515.324 nm, Cu(i) 521.820 nm, Cu(i) 529.252 nm, Cu(i) 570.024 nm and Cu(i) 578.213 nm, the plasma electron density values were calculated to be 2.112 × 10^16^ cm^−3^, 0.776 × 10^16^ cm^−3^, 0.847 × 10^16^ cm^−3^, 1.406 × 10^16^ cm^−3^, 1.739 × 10^16^ cm^−3^, and 1.158 × 10^16^ cm^−3^, respectively, which range from approximately 0.776 × 10^16^ cm^−3^ to 2.112 × 10^16^ cm^−3^. The calculation error is approximately ±25%, consisting mainly of the parameter errors in the electron density calculation, the measurement errors in the spectral data, the half-width errors obtained from data fitting and the instrumental measurement errors. The plasma electron density is determined from numerical analysis to be 1.724 × 10^16^ cm^−3^, which is within the range found from the experimental results.

In summary, the plasma electron temperature is approximately 8438 K, the electron density is approximately 0.776 × 10^16^ cm^−3^ to 2.112 × 10^16^ cm^−3^, and the numerical analysis results are within the range obtained from the experimental results, which also verifies the correctness and feasibility of the plasma expansion model.

#### 4.2.3 Plasma energy absorptivity

In the experiments, an infrared laser operating at a wavelength of 1064 nm was used. The literature^8^ shows that the plasma formed during infrared laser processing of metals absorbs energy mainly controlled by the inverse bremsstrahlung absorption effect. The main process involves the reionization or secondary ionization of free electrons in the ion Coulomb field due to the absorption of laser energy and the accelerating of its transfer to atoms or ions. The inverse bremsstrahlung absorption is mainly expressed as the absorption coefficient *α*_ib_:^31^30
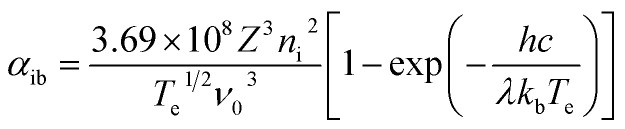


**Fig. 11:**
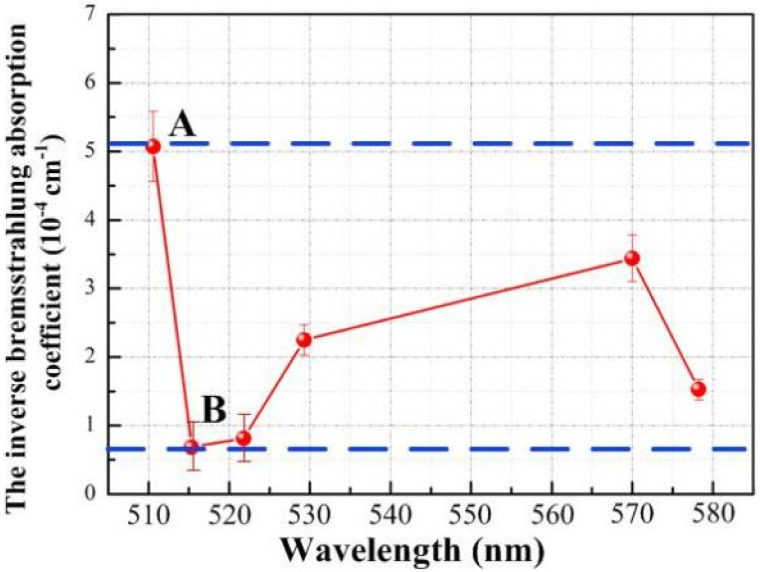
Evolution curve of the plasma inverse bremsstrahlung absorption coefficient.

The plasma energy absorptivity can be calculated from the inverse bremsstrahlung absorption coefficient, that is, the value of the plasma shielding effect. The plasma electron temperature and plasma electron density calculated from six copper atomic spectral lines was substituted into eqn (30) to obtain the evolution curve for the plasma inverse bremsstrahlung absorption coefficient, as shown in Fig. 11. The evolution law is similar to the plasma electron density distribution, with a maximum and minimum absorption coefficient value of approximately 5.073 × 10^−4^ cm^−1^ at point A and approximately 0.684 × 10^−4^ cm^−1^ at point B, respectively. Based on the laser transmittance formula exp(−*α*_ib_*L*), the laser transmittance was calculated to be approximately 100%. Therefore, the plasma energy absorption rate is extremely low and can be neglected in practical processing.

The above calculations show that the plasma expansion tends to a Maxwell distributed equilibrium state after approximately 2 μs. When the laser processing parameters are lower than the experimental conditions (laser wavelength of 1064 nm, pulse frequency of 20 kHz, pulse width of 210 ns, laser power density of 3.359 × 10^8^ W cm^−2^), the laser energy loss due to plasma inverse bremsstrahlung is negligible.

### 4.3 Grinding experiments

**Fig. 12:**
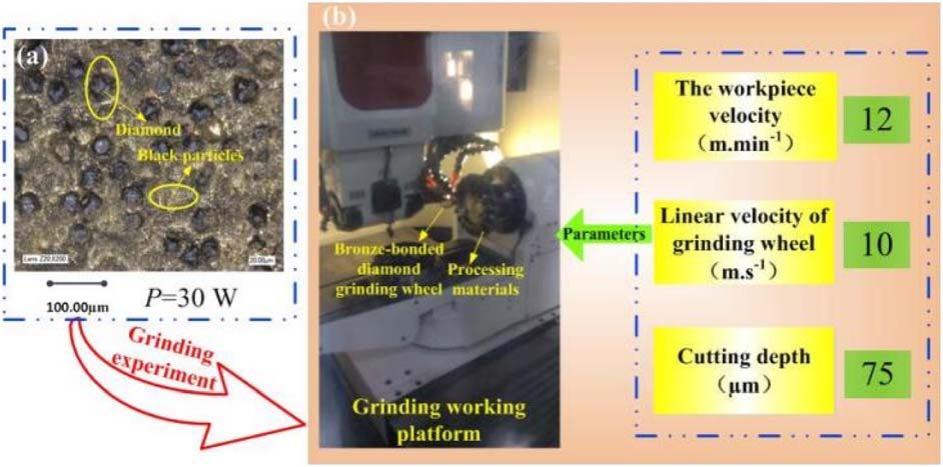
Bronze diamond grinding wheel and grinding platform and grinding parameters. (a) Topography of the bronze diamond grinding wheel; (b) grinding platform and grinding parameters.

Before the grinding experiments, the surface profile of the grinding wheel after laser processing was observed by a three-dimensional ultradeep field microscope system. Fig. 12(a) shows the topography of the bronze diamond grinding wheel. The diagram shows a large number of black particles covering the bronze surface and around the diamond abrasive grains. This is due to the plasma formed on the surface of the grinding wheel during the laser process, where the combination of copper ions with oxygen will generate black copper oxide particles, which collide during the expansion process, leading to a change in the velocity direction and a return to cover the surface of the bronze grinding wheel. The grinding platform and grinding parameters are shown in Fig. 12(b). The grinding wheel was fixed to the grinding machine, and cemented carbide was used as the grinding material.

**Fig. 13:**
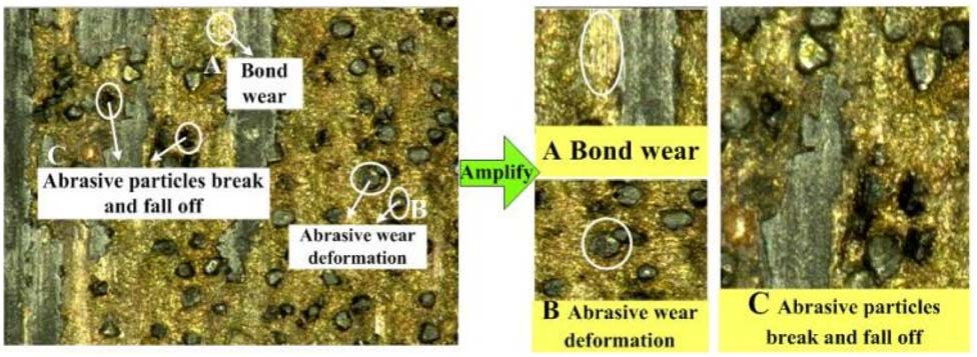
Topography of the bronze diamond grinding wheel.

Grinding studies have shown^25^ that grinding wheels lose their grinding performance during the grinding process mainly due to wear due to their own abrasion and external crushing. In self-wear, the abrasive grain is in direct contact with the surface of the material being ground. During the grinding process, high temperatures are formed at the grinding site, resulting in chemical changes, and the abrasive grains are repeatedly scratched, squeezed and pressed against the material surface, resulting in their own plastic deformation. The grinding edge, the tip of the abrasive grain, the surface of the abrasive grain and other parts are worn down to flatness and roundness, therefore leading to a loss of grinding performance. External breakage mainly refers to the contact between the material being ground and the bond or abrasive grain. During the grinding process, the temperature and grinding force change. At the same time, the high contact stress intensity leads to the bond wearing away or the abrasive grains to fracture and fall off, resulting in breakage of the grinding contact area making subsequent grinding impossible. The surface quality of the grinding wheel after grinding was photographed at a magnification of 200 times under a three-dimensional ultradeep field microscope system, as shown in Fig. 13.

The bond surface shown in Fig. 13A is flat, which is mainly due to the bond and the grinding material rubbing against each other and the bond wearing down to expose a new surface for a higher grinding force. The abrasive grains shown in Fig. 13B undergo wear deformation, forming a more rounded plane with essentially no grinding edge. The abrasive grains shown in Fig. 13C are fractured and dislodged, and the shapes of the abrasive grains are fundamentally deformed, which leads to an increase in the surface roughness of the ground material.

**Fig. 14:**
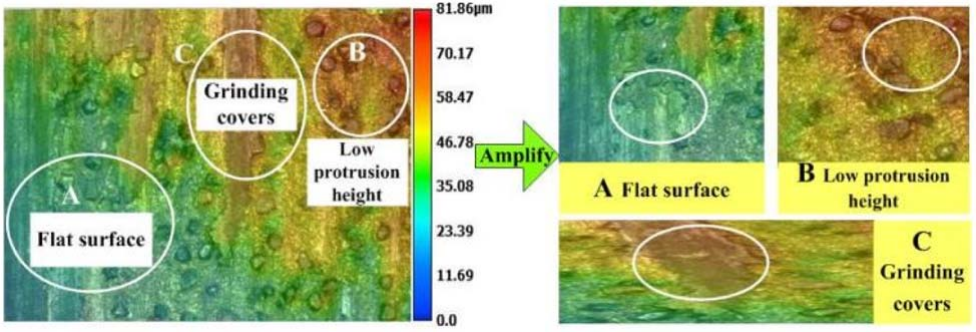
Layered topography of the grinding wheel.

Fig. 14 shows the layered topography of the grinding wheel, with the highest areas of the protruding surface shown in red and the lowest areas shown in blue. The gradual evolution of the graphic colour markings from blue to red indicates the evolution law for the protrusion height, which in turn can be used to characterize the topography of the pitted and undulating surface of the grinding wheel. The maximum protrusion height value in the red part of the diagram is approximately 81.86 μm. However, there are fewer red areas and more light green areas, as shown in Fig. 14A, with protrusion heights of approximately 35 μm–45 μm, all with low protrusion heights. As shown in Fig. 14B, although the abrasive grains protrude through the bond, the protrusion height is low, approximately 45 μm–56 μm, and a poor regrinding performance for the grinding wheel is obtained after grinding, whereas, as shown in Fig. 14C, the grinding wheel surface is covered with a thick layer of grinding material, which wraps all the abrasive grains.

Fig. 13 and 14 show that the surface of the laser processed grinding wheel is covered with returning black particles, which reduces the height of the grinding grain protruding from the bond, causing the bond to participate in the grinding, wrapping the grinding edge, blocking the chip space around the abrasive grains. This results in a reduction in the number of abrasive grains directly involved in grinding, making the grinding process mainly dependent on the extrusion of the diamond itself to remove material, causing the abrasive grains to break up, producing a flattening effect and reducing its grinding performance.

## 5 Conclusion

In this paper, plasma characteristic models in cylindrical coordinates were established and applied to numerically analyse the plasma-related characteristics of pulsed laser processing of bronze-diamond grinding wheels. The main conclusions are as follows:

(1) The plasma expansion models for pulsed laser processing of materials in cylindrical coordinates were established by considering the Maxwell velocity distribution characteristics, and the change rate model of the plasma collisional ionization was obtained by coupling the two-body collision frequency equation. The model has the advantages of a simple structure and convenient solution, which provides theoretical guidance for further research on the plasma characteristics of laser processing of metal materials.

(2) Numerical analysis shows that the plasma expansion velocity and expansion dimensions in the laser processing of a bronze-diamond grinding wheel gradually increase with time, the concentration spatial distribution and expansion-induced external pressure distribution shows high inside and low outside characteristics, the collision change rate is large, and under the thermal effect, the formation of black copper oxide particles is followed by their return to cover the surface of the material. The mechanism for particle deposition effects in laser processing is revealed based on models, simulations and experiments, which can also be used to study particle deposition effects in LIBS and PLD, with some practical implications.

(3) The plasma emission spectrum for laser processing of bronze-diamond grinding wheels was measured, and calculations show that the average plasma temperature is approximately 8500 K and average plasma electron density ranges from approximately 0.776 × 10^16^ cm^−3^ to 2.112 × 10^16^ cm^−3^. The plasma shows no shielding effect and the phenomenon of a large number of black particles covering the surface of the grinding wheel after processing is observed. The grinding experiments show that the covered particles reduce the processing quality and affect the grinding performance. The experiments not only verify the correctness and feasibility of the plasma characteristic models but also provide theoretical guidance and process optimization for subsequent research on laser processing of materials.

## Appendix

### 1 Plasma concentration spatial distribution model and plasma expansion kinetic model

The plasma distribution equation in cylindrical coordinates is established as follows:31
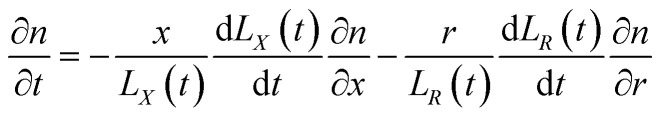


In the *X*-direction, the relationship is as follows:32
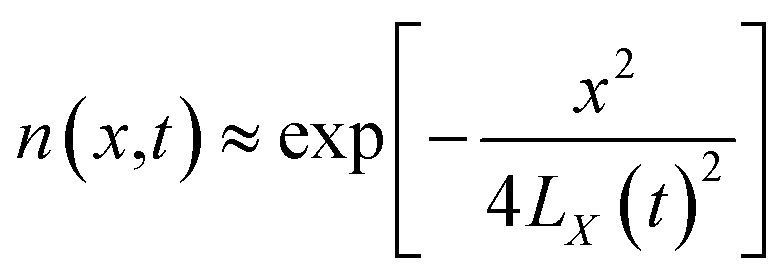


In the *R*-direction, the relationship is as follows:33
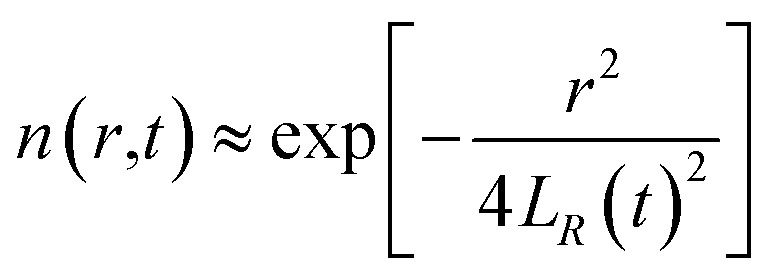


The plasma concentration spatial distribution equation can be solved to obtain:34
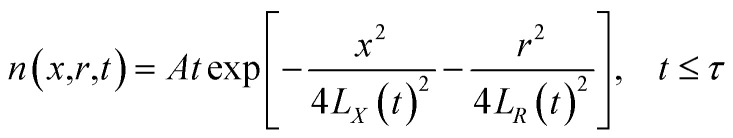


The integration in cylindrical coordinates can be obtained as follows:35
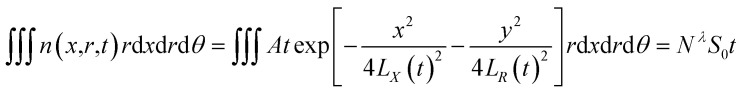
36
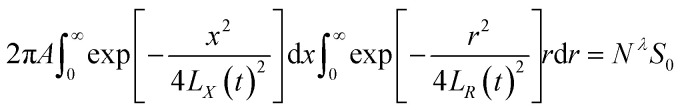


The relationship is: 
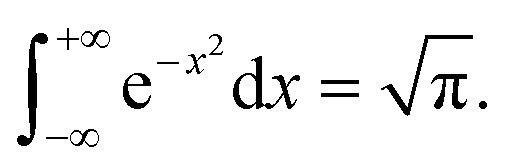
 The expression for *A* and *N*^*λ*^ can be found from the literature:37
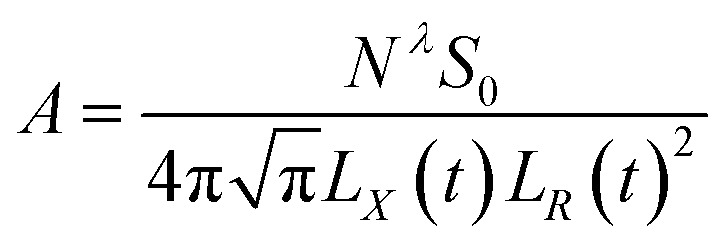
38
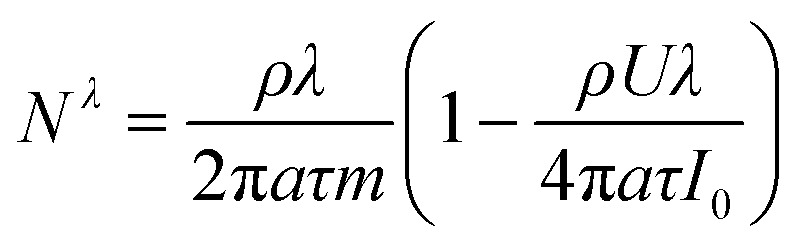


By substituting eqn (37) and (38) into eqn (34), one can be obtained the plasma concentration spatial distribution equation:39
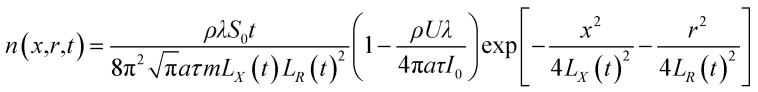


Considering the equation *P* = *nk*_b_*T* under ideal gas conditions, the plasma expansion-induced external pressure equation is obtained as follows:40
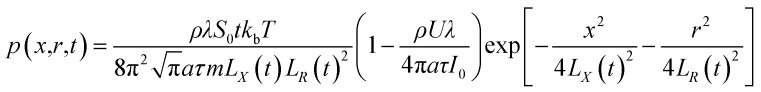


In cylindrical coordinates, the local mass conservation law can be expressed as follows:41
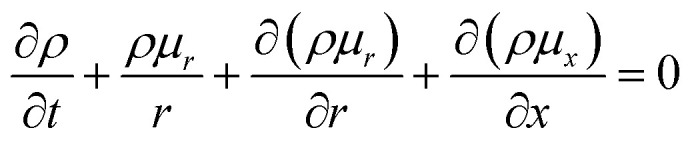


By substituting the spatial distribution of the plasma concentration into eqn (41), we can obtain:42
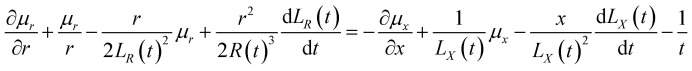


Eqn (42) requires both expressions on either side of the equal sign to be constant; so, both sides of the equation must be a function of time *t*(*q*(*t*)), and we can obtain:43
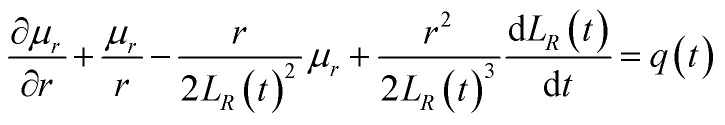
44
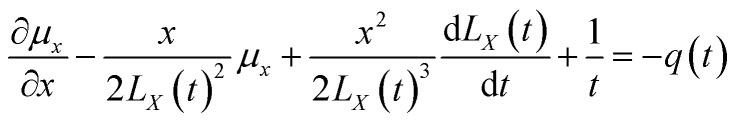


Eqn (43) can be solved to obtain:45
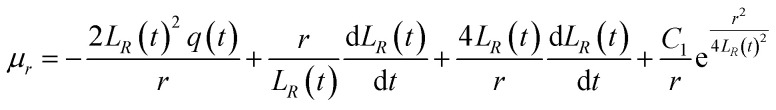


Eqn (44) can be solved to obtain:46
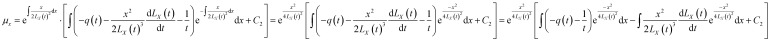


By using the integration formula for exponential functions:47
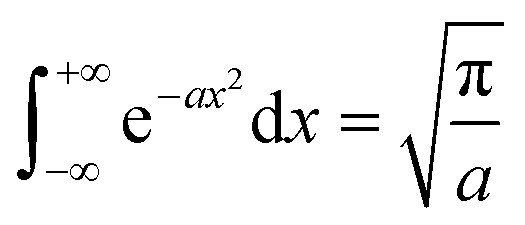


We can obtain48
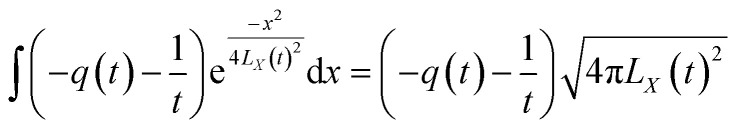


By using the Gauss integration formula:49
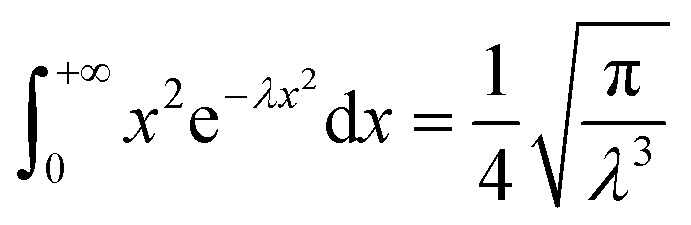


We can obtain50
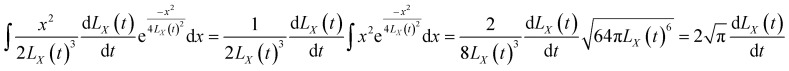


By substituting eqn (48) and (49) above into eqn (46), we can obtain51
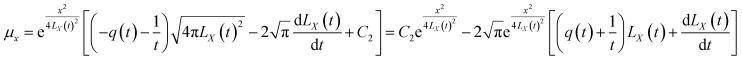
where the constants *C*_1_ and *C*_2_ were determined by the following boundary conditions gives:
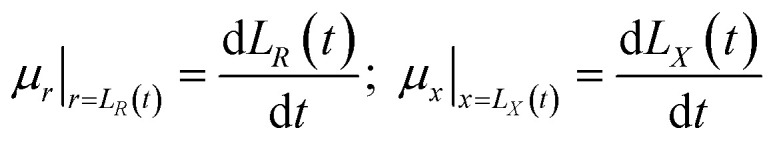


From the above boundary conditions, we can obtain52
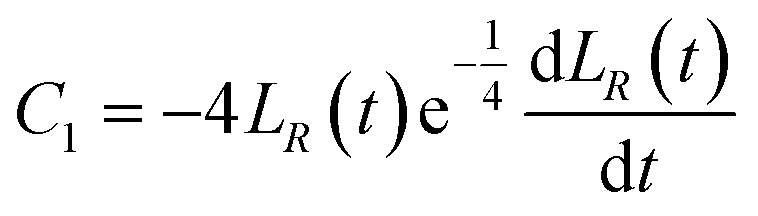
53
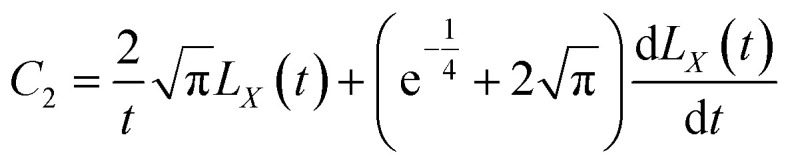


By substituting eqn (52) and (53) into eqn (45) and (51), respectively, we can obtain54
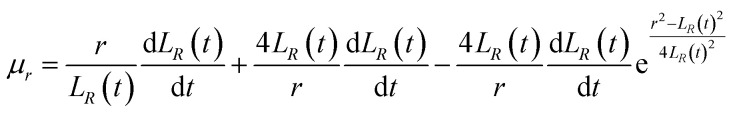
55
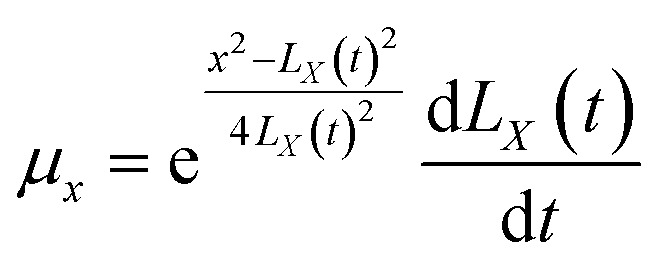


Eqn (54) and (55) can be used to derive for time (*t*) and position (*x*) respectively:56
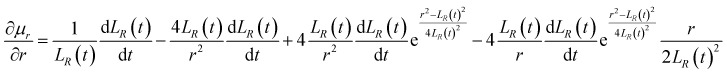
57
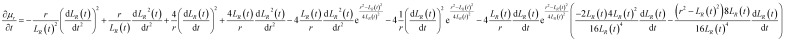
58
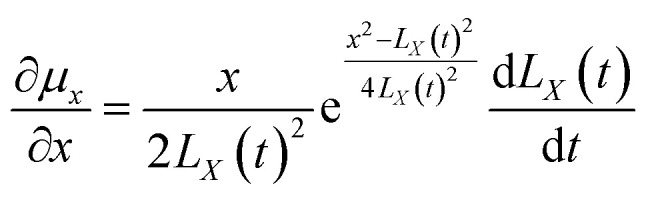
59
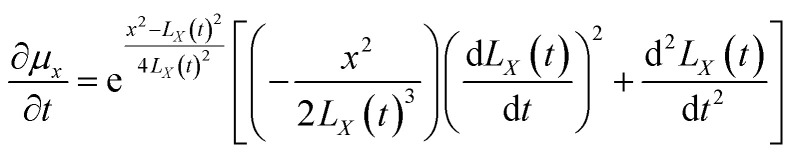


In cylindrical coordinates, the conservation of momentum equations are as follows:60
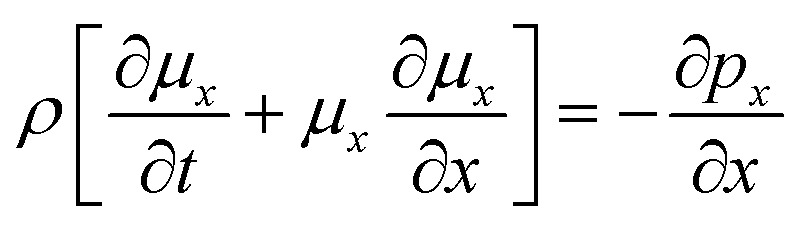
61
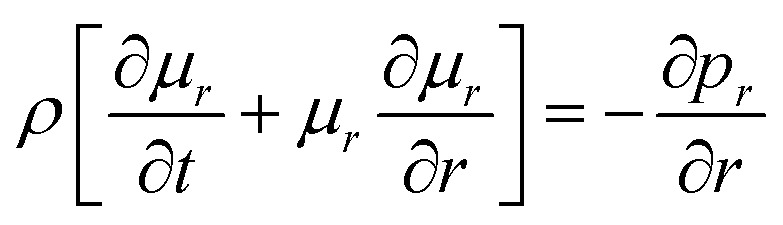


By substituting *x* = *L*_*X*_(*t*) and *r* = *L*_*R*_(*t*) into eqn (56)–(59), the plasma expansion kinetic equations are obtained as follows:62
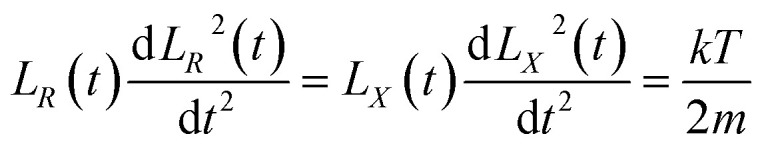
*T* is the plasma electron temperature, K, where the initial velocity is given by:63
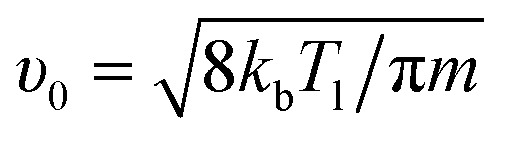


### 2 Change rate model of the plasma collisional ionization effect

The collision cross section between the electron and neutral particle is given by:

64 *σ* = π*r*^2^

The average molecular free path is calculated to be:

65 *λ* = 1/(*nσ*)

The average plasma electron velocity is expressed as follows:66
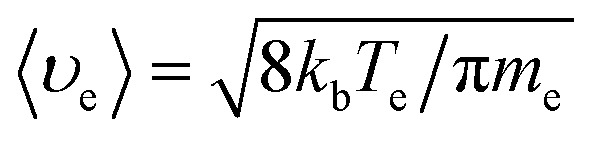


According to the two-body collision frequency equation:67
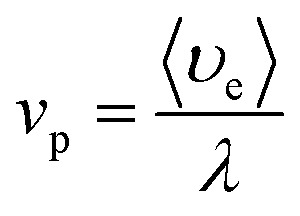


By substituting the equation for the average free path into the plasma concentration spatial distribution equation, the change rate equation for the plasma collisional ionization is obtained as follows:68
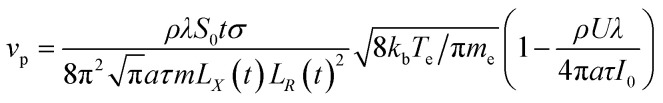


## Author contributions

Conceptualization, Song Cai; methodology, Song Cai; model derivation, Wenhao Liu; software, Song Cai and Wenhao Liu; writing-original draft preparation, Song Cai; writing-review and editing, Juan Wang; visualization, Juan Wang; experimental validation, Juan Wang and Qi Li; formal analysis, Wenhao Liu; data curation, Wenhao Liu; resources, Qi Li and Zheying Zhang; project administration, Guoqi He and Yi Ji; funding acquisition, Guoqi He and Yi Ji; investigation, Zheying Zhang; supervision, Yi Ji. All authors have read and agreed to the published version of the manuscript.

## Conflicts of interest

There are no conflicts to declare.

## Nomenclature

*L*_*X*_(*t*): the edge space dimensions at the plasma expansion time *t* in the *X*-direction [m]
*L*_*R*_(*t*): the edge space dimensions at the plasma expansion time *t* in the *R*-direction [m]
*n*(*x*,*t*): the plasma electron density at the plasma expansion time *t* in the *X*-direction [m^−3^]
*n*(*r*,*t*): the plasma electron density at the plasma expansion time *t* in the *R*-direction [m^−3^]
*n*(*x*,*r*,*t*): the plasma electron density at the plasma expansion time *t* [m^−3^]
A
: the normalized parameter
*N* ^*λ*^
: the laser ablation efficiency
*S*_0_
: the spot size [m^2^]
t
: the ablation time [s]
α
: the refractive index of the material
U
: the excitation energy [eV]
τ
: the pulse time [s]
m
: the atomic mass [kg]
*I*_0_
: the laser power density [W cm^−2^]
P
: the plasma expansion-induced external pressure [Pa]
*k*_b_
: the Boltzmann constant [1.381 × 10^−23^ J K^−1^]
T
: the plasma electron temperature [K]
*μ*_*r*_
: the velocity parallel to the material surface [m s^−1^]
*μ*_*x*_
: the velocity perpendicular to the material surface [m s^−1^]
*υ*_0_
: the initial velocity [m s^−1^]
σ
: the particle collision cross section
*L*_*z*_
: the average molecular free path [m]
〈*υ*_e_〉: the average plasma electron velocity [m s^−1^]
*v*_p_
: the change rate equation of the plasma collisional ionization [s^−1^]
*m*_e_
: the electron mass [kg]
*T*_l_
: the material gasification temperature [K]
ρ
: the material solid phase density [kg m^−3^]
*k*_d_
: the thermal conductivity [W (m K)^−1^]
η
: the laser absorptivity
b
: the thermal diffusivity [cm^2^ s^−1^]
*l*_m_
: the pulse width [s]
*T*_v_
: the gasification temperature [K]
*T*_0_
: the initial temperature [K]
*n*_i_
: the ion number density [cm^−3^]
*T*_e_
: the plasma electron temperature [K]
*ν*_0_
: the incident light frequency [Hz]
Z
: the average electric charge [C]

## Supplementary Material
